# Supplementary material for: Benchmarking automated cell type annotation tools for single-cell ATAC-seq data
Source: Front Genet. 2022 Dec 13;13:1063233. doi: 10.3389/fgene.2022.1063233 (PMC9792779; doi:10.3389/fgene.2022.1063233)
Supplement: Supplementary file 1 [file DataSheet1.docx]

# Supplementary Figures


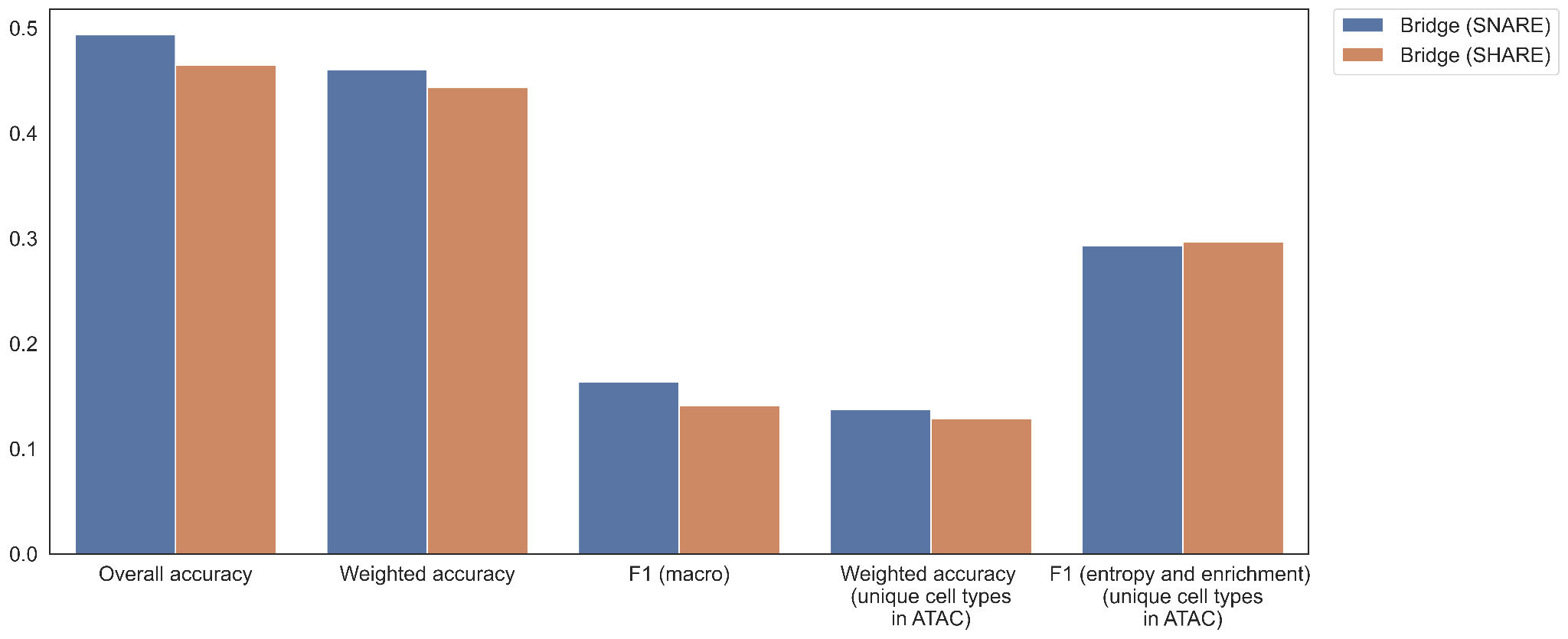


**Supplementary Figure 1.** The performance of Bridge integration on the mouse brain data with SNARE-seq or SHARE-seq data as the ‘bridge’.


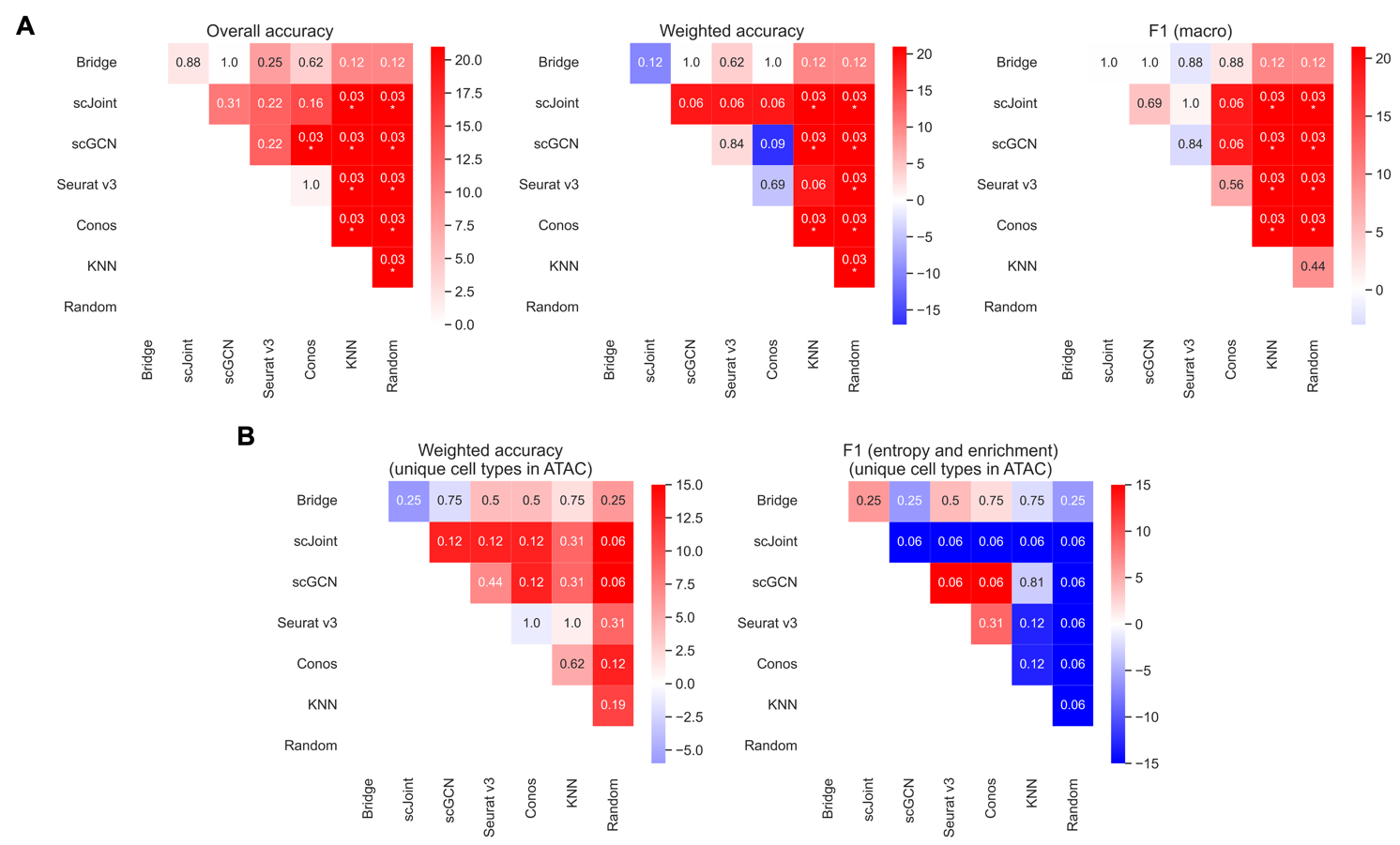


**Supplementary Figure 2.** Heatmaps showing the paired Wilcoxon signed-rank test results between any two methods across all tissues for the three overall metrics (A) and two metrics for ATAC-specific cell types (B). The color bar represents the statistics of Wilcoxon signed-rank tests, which were calculated based on subtracting the metric values of the method in the column from that of the method in the row. Red colors indicate that the method in the row had overall higher scores than the method in the column, while blue colors suggest the opposite direction. Values shown on the heatmaps are nominal p-values and those less than 0.05 were annotated with asterisks (*). In (A), for comparisons that involved Bridge, only four data points were available (brain, kidney, BMMC and PBMC); while for other comparisons, six data points were used (two additional were lung (FACS) and lung (droplet)). In (B), for comparisons that involved Bridge, only three data points were available (brain, kidney and PBMC); while for other comparisons, five data points were used (two additional were lung (FACS) and lung (droplet)).


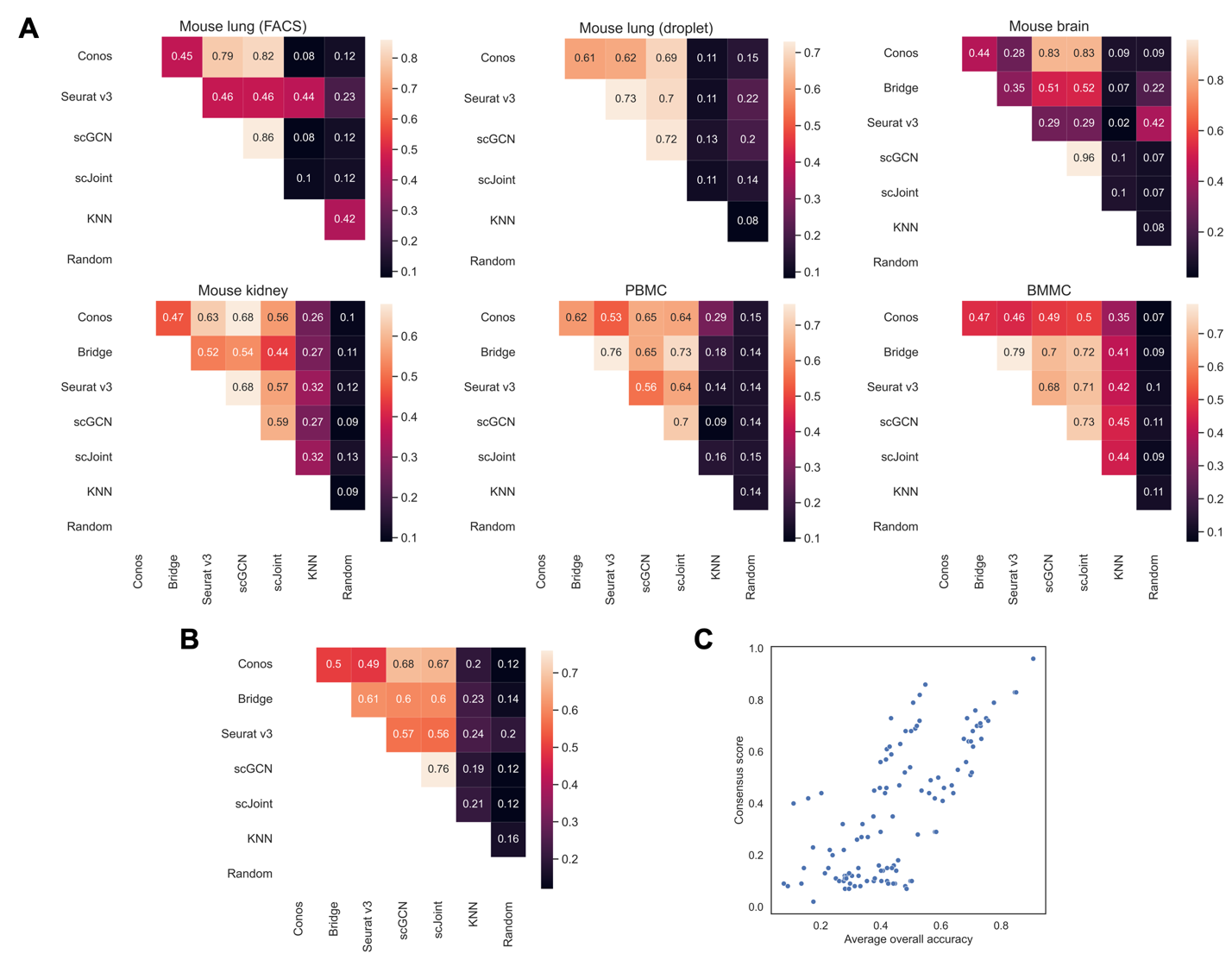


**Supplementary Figure 3.** Consensus analysis among methods measured by the proportion of ATAC cells that were equally annotated. (A) Heatmaps showing the results in each tissue. (B) Heatmap showing the overall consensus results by averaging across tissues. (C) Strong positive correlation between consensus scores and average overall accuracy between any two methods.


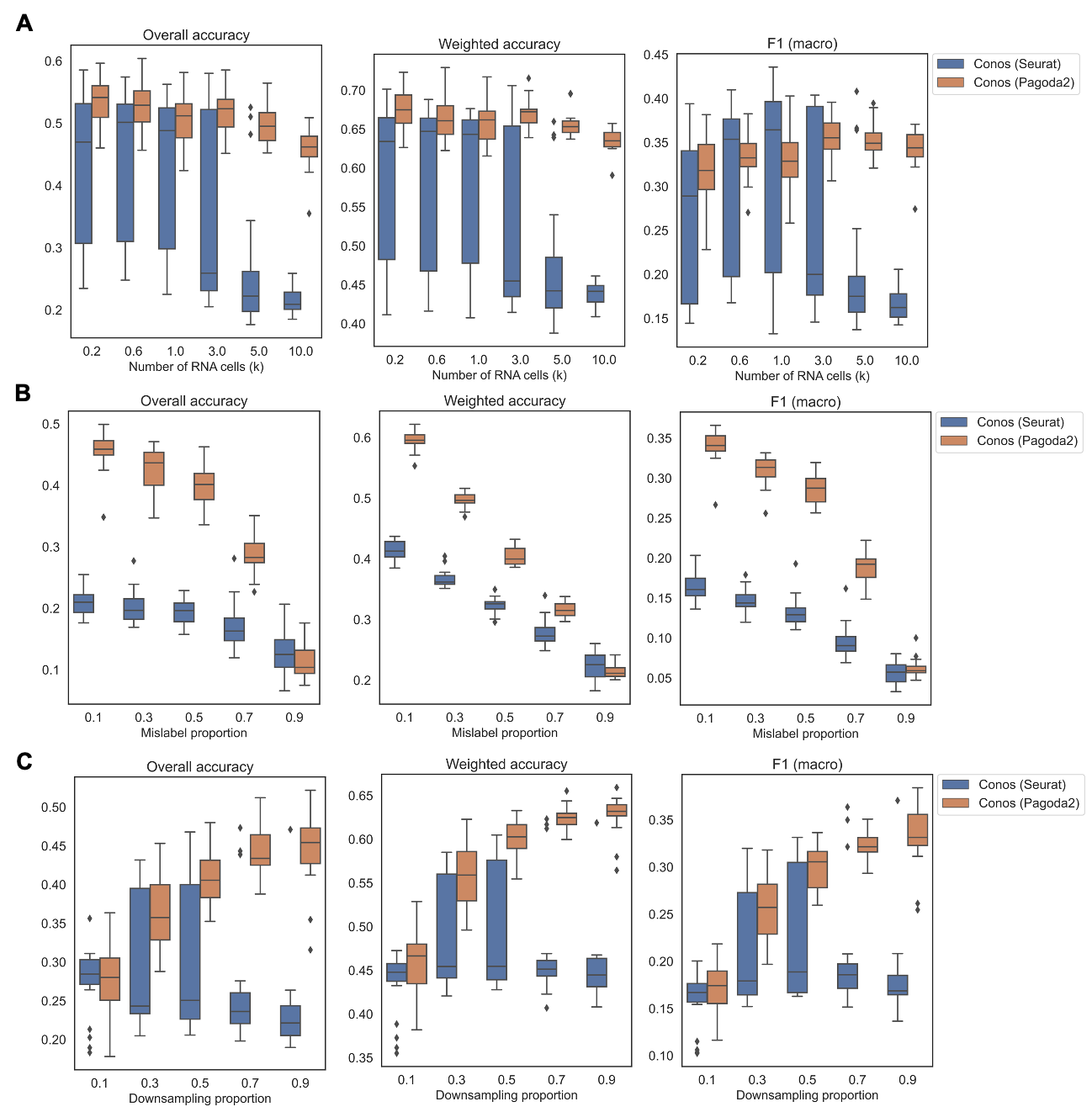


**Supplementary Figure 4.** Comparison of Conos combined with data processing pipeline by Seurat or Pagado2 across different BMMC experiment settings presented in the main text. Central lines represent medians, boxes represent the interquartile range (IQR), and the upper/lower whisker represents the largest/smallest value no further than 1.5 × IQR.


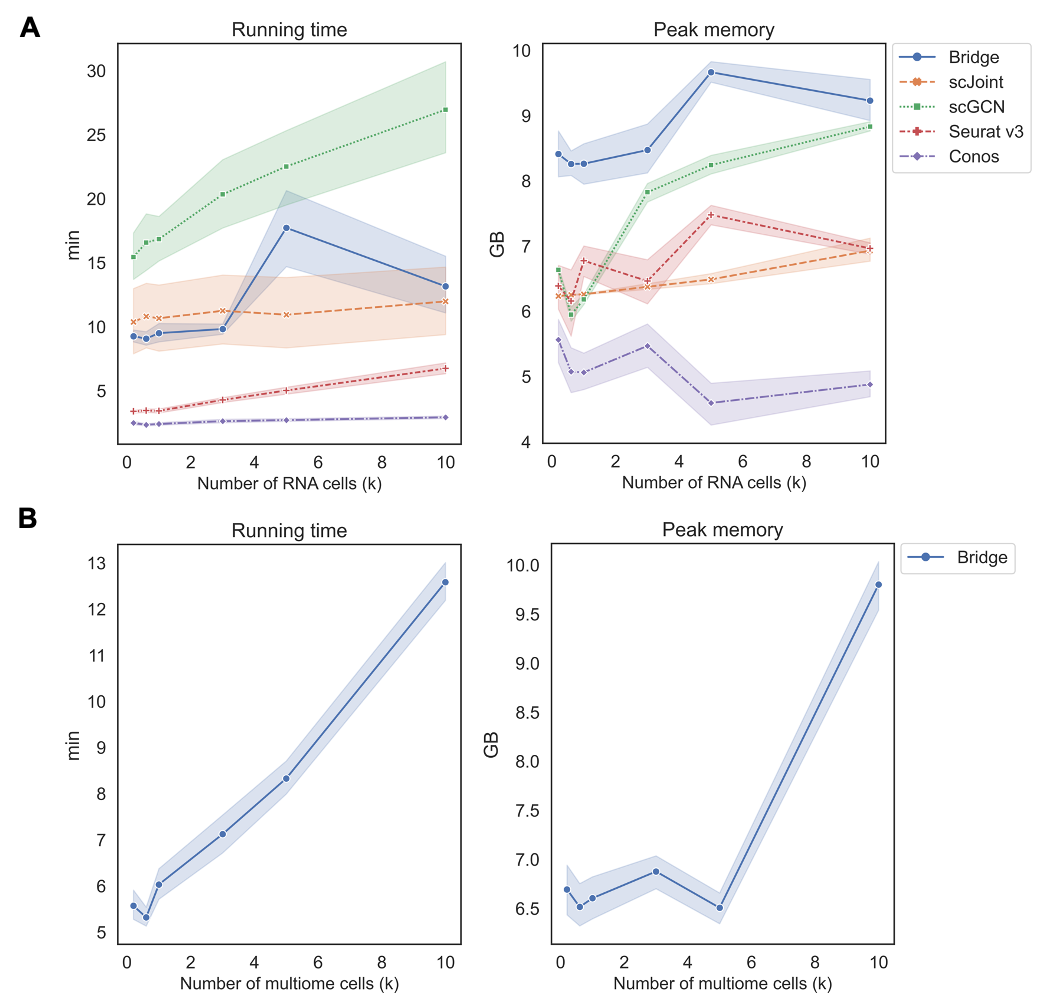


**Supplementary Figure 5.** Running time and peak memory usage of methods when changing number of RNA cells (A) and when changing number of multimodal cells (for Bridge only) (B). The error band shows the 95% confidence interval.


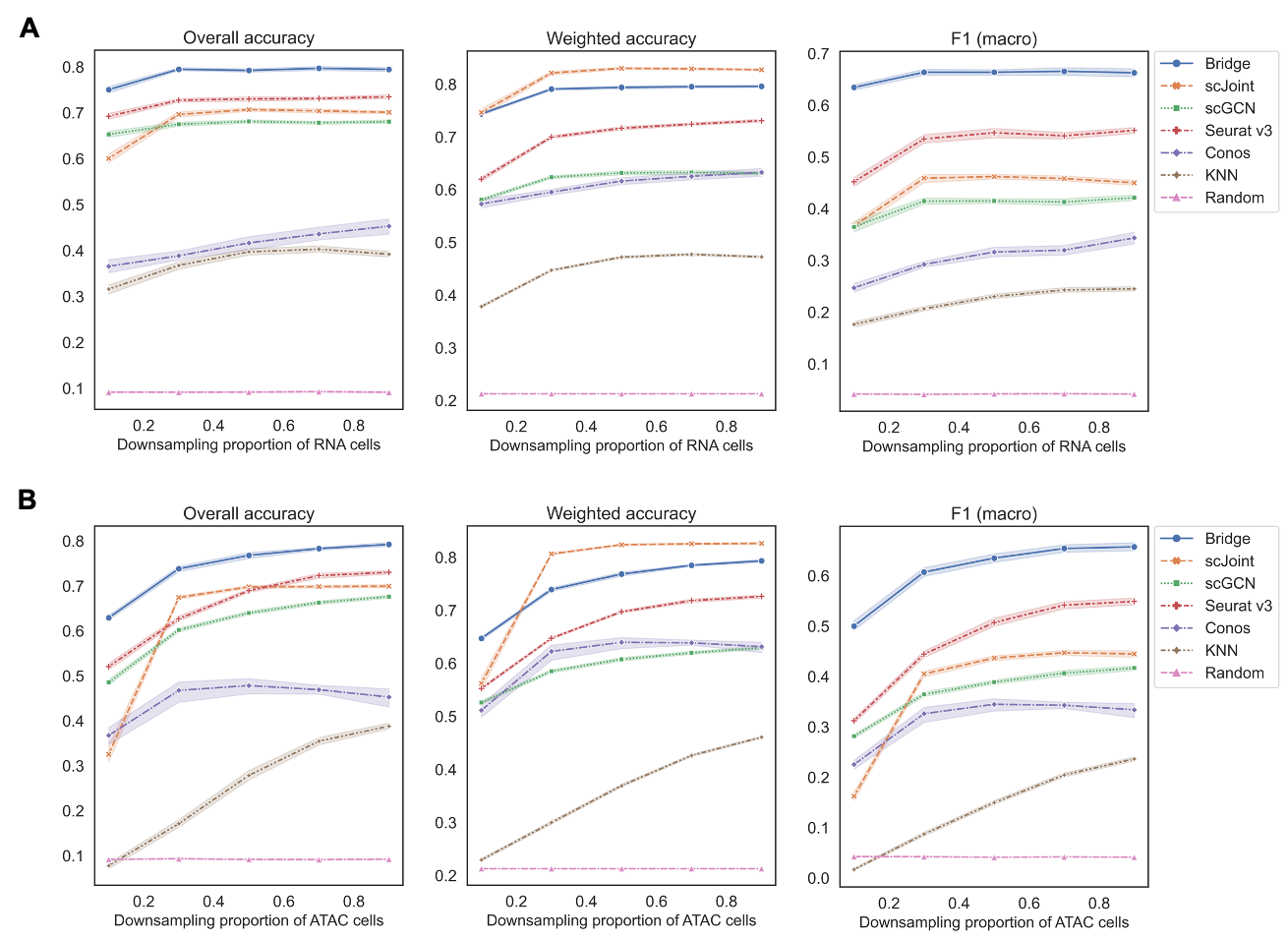


**Supplementary Figure 6.** Performance of methods on different downsampling proportions of scRNA-seq (A) and scATAC-seq (B) only, respectively. The error band shows the 95% confidence interval.


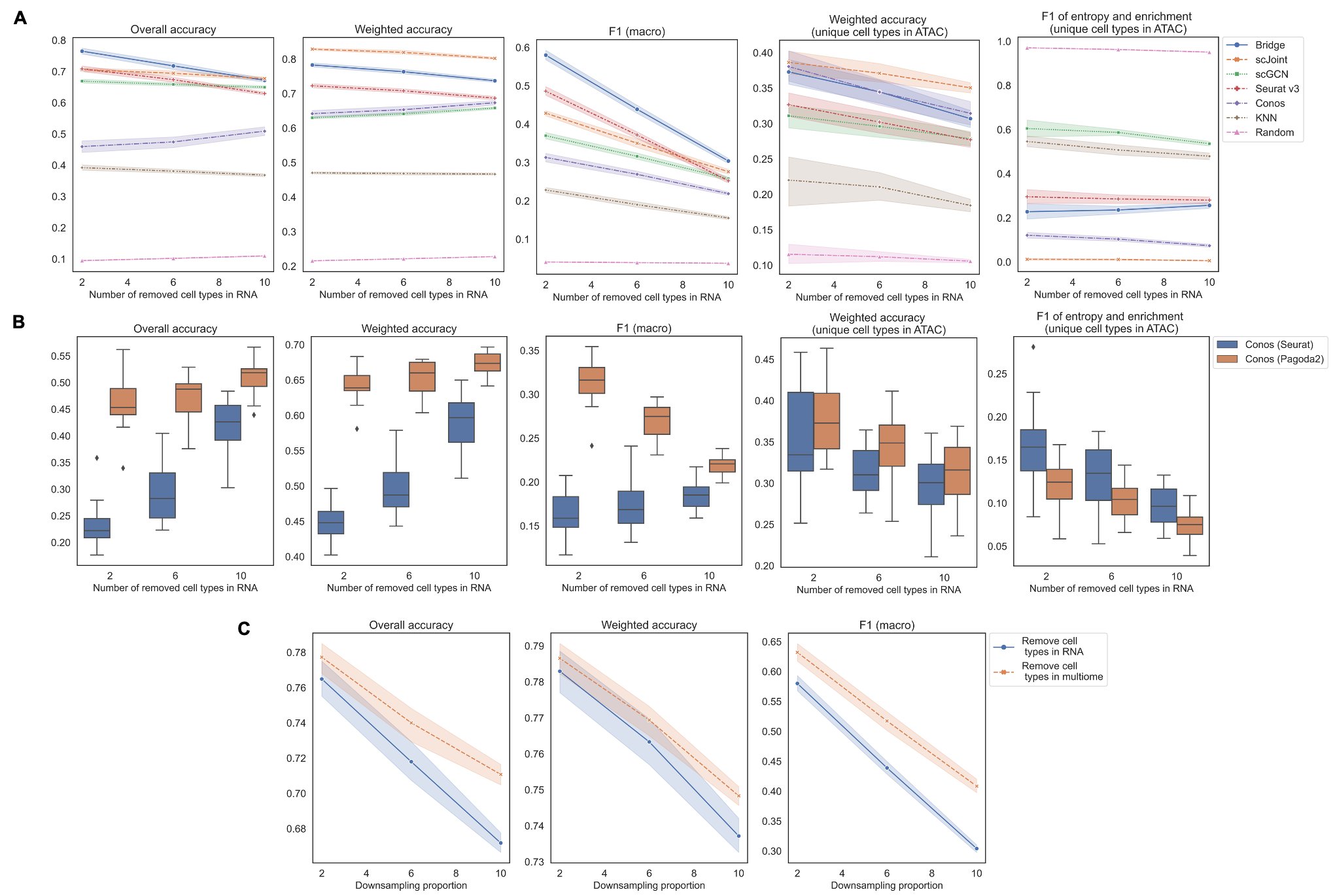


**Supplementary Figure 7.** Performance of methods on different numbers of removed cell types. (A) Remove cell types in labeled RNA data to make corresponding cell types in ATAC data unique. (B) Performance of Conos using either Seurat or Pagoda2 for data processing. Central lines represent medians, boxes represent the interquartile range (IQR), and the upper/lower whisker represents the largest/smallest value no further than 1.5 × IQR. (C) Performance of Bridge when removing cell types in RNA or multimodal data, respectively. The error band shows the 95% confidence interval.
